# Some notes about one inequality with power functions

**DOI:** 10.1186/s13660-018-1780-1

**Published:** 2018-07-21

**Authors:** Ladislav Matejíčka

**Affiliations:** Faculty of Industrial Technologies in Púchov, Trenčín University of Alexander Dubček in Trenčín, Púchov, Slovakia

**Keywords:** 26D15, Inequalities with power functions, Power mean

## Abstract

In this paper, we prove one inequality with power functions. A simplified form of the inequality was published as the problem 12024-02 in the American Mathematical Monthly.

## Introduction

Inequalities with power functions have many important applications. They can be found in mathematical analysis and in other theories like ordinary differential equations, probability theory and statistics, chemistry, economics, mathematical physics, and mathematical biology. Not long ago, the following problem (1) was published in the AMM [2].

Problem 12024-02- M. Cucoanes, M. Dragan, and N. Stanciu (Romania).

Let *x*, *y*, and *z* be positive real numbers satisfying $xyz=1$. Prove

1 $$ \bigl(x^{10}+y^{10}+z^{10} \bigr)^{2}\geq 3 \bigl(x^{13}+y^{13}+z^{13} \bigr). $$

The aim of this paper is to prove a more general form of inequality (1). We also discuss other forms of (1).

## Methods

In this paper, methods of mathematical and numerical analysis are used. We use also the software MATLAB for some computing.

## Results and discussion

In this section we prove a more general form of (1).

### Lemmas and theorems

#### Lemma 1

*Let*

$$\begin{aligned} \begin{gathered} g(x_{1},\ldots,x_{n}) = \Biggl( \sum_{i=1}^{n}x_{i}^{a} \Biggr)^{m}-q \Biggl(\sum_{i=1}^{n}x_{i}^{b} \Biggr)\quad \textit{where } n\in N; n\geq3; m>0; \\ \quad q,a,b>0; a\neq b; \prod_{i=1}^{n}x_{i}=1; 0< x_{1}< \cdots< x_{n}. \end{gathered} \end{aligned}$$

*Then* *g* *has no local extremes in*

$$W= \Biggl\{ (x_{1},\ldots,x_{n}); 0< x_{1}< 1, x_{1}< x_{2}< \cdots< x_{n}, \prod _{i=1}^{n}x_{i}=1 \Biggr\} . $$

#### Proof

Put

$$\begin{aligned} h =& \Biggl(\sum_{i=1}^{n-1}x_{i}^{a}+ \frac{1}{\prod_{i=1}^{n-1}x_{i}^{a}} \Biggr)^{m}-q \Biggl(\sum _{i=1}^{n-1}x_{i}^{b}+ \frac{1}{\prod_{i=1}^{n-1}x_{i}^{b}} \Biggr). \end{aligned}$$

We have

$$\begin{aligned} h'_{x_{i}} =& ma \Biggl(\sum_{i=1}^{n}x_{i}^{a} \Biggr)^{m-1} \biggl(x_{i}^{a-1}-\frac{1}{x_{i}\prod_{i=1}^{n-1}x_{i}^{a}} \biggr) -qb \biggl(x_{i}^{b-1}-\frac{1}{x_{i}\prod_{i=1}^{n-1}x_{i}^{b}} \biggr) \end{aligned}$$

for $i=1,\ldots,n-1$. If $h'_{x_{i}}(x_{1},\ldots,x_{n-1})=0$ for $i=1,\ldots,n-1$ for some $(x_{1},\ldots,x_{n-1})\in W$, then we have

$$\begin{aligned} \frac{x_{i}^{a}-x_{n}^{a}}{x_{i}^{b}-x_{n}^{b}} =& \frac {x_{j}^{a}-x_{n}^{a}}{x_{j}^{b}-x_{n}^{b}}\quad\text{for } i,j=1,\ldots,n-1, i\neq j. \end{aligned}$$

It implies

$$\begin{aligned} \frac{ (\frac{x_{i}}{x_{n}} )^{a}-1}{ (\frac {x_{j}}{x_{n}} )^{a}-1} =& \frac{ (\frac{x_{i}}{x_{n}} )^{b}-1}{ (\frac{x_{j}}{x_{n}} )^{b}-1}\quad\text{for } i,j=1,\ldots,n-1, i\neq j. \end{aligned}$$

We show that

$$ f(t)=\frac{t^{a}-1}{t^{b}-1} $$

is a strictly monotonic function for $t\in(0,1)$ and $a\neq b$, $a,b>0$.

We have

$$ f'_{t}=\frac{s(t)}{(1-t^{b})^{2}}=\frac {(a-b)t^{a+b-1}-at^{a-1}+bt^{b-1}}{(1-t^{b})^{2}}. $$

If $0< a< b$, then $s(t)<0$.

Really, from $s(t)=t^{a-1}w_{1}(t)=t^{a-1}((a-b)t^{b}-a+bt^{b-a})$. Because of $w_{1}(1)=0$ and $w_{1t}'=b(b-a)t^{b-a-1}(1-t^{a})>0$, we obtain $f(t)$ is a strictly decreasing function on $(0,1)$.

If $0< b< a$, then $s(t)>0$.

Really, we have $s(t)=t^{a-1}w(t)=t^{a-1}(-a+(a-b)t^{b}+bt^{b-a})$. Because of $w_{1}(1)=0$ and $w'_{1t}=b(b-a)t^{b-a-1}(1-t^{a})<0$, we obtain $f(t)$ is a strictly increasing function on $(0,1)$. So the proof of the lemma is complete. □

#### Lemma 2

*Let* $0< x_{i}$ *for* $i=1,\ldots,n$ *and* $\prod_{i=1}^{n}x_{i}=1$, $n\in N$, $m\geq1$, $k,a>0$. *Then*

$$\begin{aligned} \Biggl(\sum_{i=1}^{n}x_{i}^{a+k} \Biggr)^{m}\geq n^{m-1} \Biggl(\sum _{i=1}^{n}x_{i}^{a} \Biggr). \end{aligned}$$

#### Proof

Put

$$\begin{aligned}& v(t) = \Biggl(\sum_{i=1}^{n}x_{i}^{t} \Biggr)^{m},\quad\text{then } v'_{t}(t)=m \Biggl(\sum_{i=1}^{n}x_{i}^{t} \Biggr)^{m-1} \times \Biggl(\sum_{i=1}^{n}x_{i}^{t} \ln(x_{i}) \Biggr)\quad\text{and} \\& \begin{aligned} v''_{tt}(t) &= m(m-1) \Biggl(\sum _{i=1}^{n}x_{i}^{t} \Biggr)^{m-2}\times \Biggl(\sum_{i=1}^{n}x_{i}^{t} \ln(x_{i}) \Biggr)^{2} +m \Biggl(\sum_{i=1}^{n}x_{i}^{t} \Biggr)^{m-1}\times \Biggl(\sum_{i=1}^{n}x_{i}^{t} \ln^{2}(x_{i}) \Biggr) \\ &\geq0. \end{aligned} \end{aligned}$$

So $v'_{t}(t)$ is an increasing function in *t*. Because of $v'_{t}(0)=0$, we obtain $v(t)$ is an increasing function in $t\geq0$. It implies

$v(a+k)\geq v(a)= (\sum_{i=1}^{n}x_{i}^{a} )^{m}$. So it suffices to show

$$ \Biggl(\sum_{i=1}^{n}x_{i}^{a} \Biggr)^{m}\geq n^{m-1} \Biggl(\sum _{i=1}^{n}x_{i}^{a} \Biggr), $$

which is evident. It follows from A–G inequality and from $\prod_{i=1}^{n}x_{i}=1$. □

#### Lemma 3

*Let* $0< x_{i}$ *for* $i=1,\ldots,n$ *and* $\prod_{i=1}^{n}x_{i}=1$, $n\in N$, $n\geq2$, $m\geq1$, $k,a>0$. *Let*

$$\begin{aligned} F= \Biggl(\sum_{i=1}^{n}x_{i}^{a} \Biggr)^{m}-n^{m-1} \Biggl(\sum_{i=1}^{n}x_{i}^{a(1+k)} \Biggr). \end{aligned}$$

*Then* $F'_{k}<0$.

#### Proof

Denote $y_{i}=x_{i}^{a}$ for $i=1,\ldots,n$. It is evident that $\prod_{i=1}^{n}y_{i}=1$. We can suppose $0< y_{i}< y_{i+1}$. So we have $0< y_{1}<1<y_{n}$. *F* can be rewritten as

$$\begin{aligned} F= \Biggl(\sum_{i=1}^{n}y_{i} \Biggr)^{m}-n^{m-1} \Biggl(\sum_{i=1}^{n}y_{i}^{(1+k)} \Biggr). \end{aligned}$$

It is evident that

$$\begin{aligned} F'_{k}=-n^{m-1} \Biggl(\sum _{i=1}^{n}y_{i}^{(1+k)} \ln(y_{i}) \Biggr). \end{aligned}$$

We show that $F'_{k}<0$ for $k>0$. $F'_{k}<0$ is equivalent to

$$\begin{aligned} S_{k}= \Biggl(\sum_{i=1}^{n}y_{i}^{(1+k)} \ln(y_{i}) \Biggr)>0. \end{aligned}$$

We have

$$\begin{aligned} S'_{k}= \Biggl(\sum_{i=1}^{n}y_{i}^{(1+k)} \ln^{2}(y_{i}) \Biggr)>0. \end{aligned}$$

The proof will be done if we show

2 $$\begin{aligned} S_{k}(k=0)= \Biggl(\sum_{i=1}^{n}y_{i} \ln(y_{i}) \Biggr)>0. \end{aligned}$$

We use the mathematical induction. For $n=2$, we get

$$\begin{aligned} S_{2}=y_{1}\ln(y_{1})+\frac{1}{y_{1}}\ln \biggl(\frac{1}{y_{1}} \biggr)=\frac{\ln(y_{1})(y_{1}^{2}-1)}{y_{1}}>0. \end{aligned}$$

Suppose that inequality (2) is valid for all $n\geq2$. We prove that (2) is valid for $n+1$. We know that $\prod_{i=1}^{n+1}y_{i}=1$. It implies

3 $$\begin{aligned} y_{1}y_{n+1}\ln(y_{1}y_{n+1})+y_{2} \ln(y_{2})+\cdots+y_{n}\ln(y_{n})>0. \end{aligned}$$

The proof will be done if we show

4 $$\begin{aligned} D=y_{1}y_{n+1}\ln(y_{1})+y_{1}y_{n+1} \ln(y_{n+1})-y_{1}\ln (y_{1})-y_{n+1} \ln(y_{n+1})< 0. \end{aligned}$$

But it is evident because of

5 $$\begin{aligned} D=y_{1}\ln(y_{1}) (y_{n+1}-1)+y_{n+1} \ln(y_{n+1}) (y_{1}-1)< 0. \end{aligned}$$

 □

#### Lemma 4

*Let* $0< x_{i}$ *for* $i=1,\ldots,n$ *and* $\prod_{i=1}^{n}x_{i}=1$, $n\in N$, $m\geq1$, $k,a>0$. *Then*

6 $$\begin{aligned} \Biggl(\sum_{i=1}^{n}x_{i}^{a} \Biggr)^{m}\leq n^{m-1} \Biggl(\sum _{i=1}^{n}x_{i}^{a(1+k)} \Biggr)\quad \textit{for } k\geq m-1. \end{aligned}$$

#### Proof

Put again $y_{i}=x_{i}^{a}$ for $i=1,\ldots,n$ and

$$\begin{aligned} F= \Biggl(\sum_{i=1}^{n}y_{i} \Biggr)^{m}-n^{m-1} \Biggl(\sum_{i=1}^{n}y_{i}^{(1+k)} \Biggr). \end{aligned}$$

We show that (6) is valid for $k=m-1$. From $F'_{k}<0$ (see previous lemma), we get (6) is valid for all $k\geq m-1$. Rewriting (6) for $k=m-1$, we obtain

$$\begin{aligned} \frac{\sum_{i=1}^{n}y_{i}}{n}\leq \biggl(\frac{\sum_{i=1}^{n}y_{i}^{m}}{n} \biggr)^{1/m}, \end{aligned}$$

which is evident because the power mean is an increasing function [1]. We note that $k=m-1$ is the best constant in (6). It follows from

$$ \lim_{y_{1}\rightarrow0^{+}} \biggl(y_{1}+n-2+\frac{1}{y_{1}} \biggr)^{m}-n^{m-1} \biggl(y_{1}^{(1+k)}+n-2+ \frac{1}{y_{1}^{(1+k)}} \biggr)\geq0 $$

for $k< m-1$.

Really,

$$\begin{aligned}& \lim_{y_{1}\rightarrow0^{+}} \biggl(y_{1}+n-2+ \frac{1}{y_{1}} \biggr)^{m}-n^{m-1} \biggl(y_{1}^{(1+k)}+n-2+ \frac{1}{y_{1}^{(1+k)}} \biggr) \\& \quad= \lim_{y_{1}\rightarrow0^{+}}\frac {(1-n^{m-1}y_{1}^{(m-1-k)})}{y_{1}^{m}}=+\infty. \end{aligned}$$

The proof is complete. □

#### Note 1

For each $a>0$ and $x_{i}>0$, $i=1,\ldots,n$, $\prod_{i=1}^{n}x_{i}=1$, $n\in N$, $n\geq2$, $m\geq1$, there is $l\geq0$ such that

7 $$ \Biggl(\sum_{i=1}^{n}x_{i}^{a} \Biggr)^{m}\geq n^{m-1} \Biggl(\sum _{i=1}^{n}x_{i}^{a(1+k)} \Biggr) $$

for all $0\leq k\leq l$, and

8 $$ \Biggl(\sum_{i=1}^{n}x_{i}^{a} \Biggr)^{m}< n^{m-1} \Biggl(\sum_{i=1}^{n}x_{i}^{a(1+k)} \Biggr) $$

for all $k>l$.

Denote, for each $m\geq1$, $n\in N$, $n\geq2$

$$ k(n,m)=\inf_{x_{i}>0, i=1,\ldots,n, \prod_{i=1}^{n}x_{i}=1}\{l\}. $$

Then $0\leq k(n,m)\leq m-1$. Next, (7) is valid for all $0\leq k\leq k(n,m)$ and for all $x_{i}>0$, $i=1,\ldots,n$, $\prod_{i=1}^{n}x_{i}=1$, and if $k>m-1$ then (8) is valid for all $x_{i}>0$, $i=1,\ldots,n$, $\prod_{i=1}^{n}x_{i}=1$. If $k(n,m)< k< m-1$, then there are some $x_{i}>0$, $i=1,\ldots,n$, $\prod_{i=1}^{n}x_{i}=1$ such that (7) is valid and there are some $x_{i}>0$, $i=1,\ldots,n$, $\prod_{i=1}^{n}x_{i}=1$ such that (8) is valid.

#### Lemma 5

*Let*

9 $$\begin{aligned} \Biggl(\sum_{i=1}^{n+1}x_{i} \Biggr)^{m}\geq(n+1)^{m-1} \Biggl(\sum _{i=1}^{n+1}x_{i}^{(1+k)} \Biggr) \end{aligned}$$

*be valid for all* $0< x_{i}$ $i=1,\ldots,n+1$ *such that* $\prod_{i=1}^{n+1}x_{i}=1$, $n\in N$, $m\geq1$, $n\geq2$, $k>0$. *Then*

10 $$\begin{aligned} \Biggl(\sum_{i=1}^{n}x_{i} \Biggr)^{m}\geq n^{m-1} \Biggl(\sum _{i=1}^{n}x_{i}^{(1+k)} \Biggr) \end{aligned}$$

*is also valid for all* $0< x_{i}$, $i=1,\ldots,n$, *such that* $\prod_{i=1}^{n}x_{i}=1$, $n\in N$, $m\geq1$, $n\geq2$, $k>0$.

#### Proof

Let $0< x_{i}$ for $i=1,\ldots,n$ and $\prod_{i=1}^{n}x_{i}=1$, $x_{n+1}=1$. Then we get

$$\begin{aligned} \Biggl(\sum_{i=1}^{n}x_{i}+1 \Biggr)^{m}\geq(n+1)^{m-1} \Biggl(\sum _{i=1}^{n}x_{i}^{(1+k)}+1 \Biggr), \end{aligned}$$

which is

$$\begin{aligned} \sum_{i=1}^{n}x_{i} \geq(n+1)^{(m-1)/m} \Biggl(\sum_{i=1}^{n}x_{i}^{(1+k)}+1 \Biggr)^{1/m}-1. \end{aligned}$$

If we show

$$\begin{aligned} (n+1)^{(m-1)/m} \Biggl(\sum_{i=1}^{n}x_{i}^{(1+k)}+1 \Biggr)^{1/m}-1\geq n^{(m-1)/m} \Biggl(\sum _{i=1}^{n}x_{i}^{(1+k)} \Biggr)^{1/m}, \end{aligned}$$

then the proof will be done. Put

$$\begin{aligned} w(t)=(n+1)^{(m-1)/m} (1+t )^{1/m}-n^{(m-1)/m}t^{1/m}-1, \quad \text{where } t=\sum_{i=1}^{n}x_{i}^{(1+k)}. \end{aligned}$$

We have

$$\begin{aligned} w'(t)=\frac{1}{m} \biggl\{ \biggl(\frac{1+n}{1+t} \biggr)^{(m-1)/m}- \biggl(\frac{n}{t} \biggr)^{(m-1)/m} \biggr\} . \end{aligned}$$

If $t< n$, then $(1+n)/(1+t)< n/t$ so $w'(t)<0$. If $t>n$, then $(1+n)/(1+t)>n/t$ so $w'(t)>0$. So $w(t)\geq w(n)=(n+1)^{m}-(n)^{m}-1$. Because of $w(n)=n^{m}(((n+1)/n)^{m}-1-1/n^{m}$, it suffices to show that $s(x)=(1+x)^{m}-x^{m}-1\geq0$ for $0\leq x\leq1$. But it follows from $s(0)=0$ and $s'(x)=mx^{m-1}((1+x)/x)^{m-1}\geq0$. The proof is complete. □

#### Note 2

We note that Lemma 5 implies $k(n+1,m)\leq k(n,m)$ for $m\geq1$. Next put

$$\begin{aligned} h= \biggl(\frac{\sum_{i=1}^{n}x_{i}}{n} \biggr)^{m}. \end{aligned}$$

Then we have

$$\begin{aligned} h'_{m}= \biggl(\frac{\sum_{i=1}^{n}x_{i}}{n} \biggr)^{m} \ln \biggl(\frac{\sum_{i=1}^{n}x_{i}}{n} \biggr)\geq0. \end{aligned}$$

So $h_{m}$ is an increasing function for *m*. From this we have: if

$$\begin{aligned} \Biggl(\sum_{i=1}^{n}x_{i} \Biggr)^{m_{1}}\geq n^{m_{1}-1} \Biggl(\sum _{i=1}^{n}x_{i}^{(1+k)} \Biggr) \end{aligned}$$

for some $m_{1}\geq1$, then

$$\begin{aligned} \Biggl(\sum_{i=1}^{n}x_{i} \Biggr)^{m_{2}}\geq n^{m_{2}-1} \Biggl(\sum _{i=1}^{n}x_{i}^{(1+k)} \Biggr) \end{aligned}$$

for $m_{2}\geq m_{1}$. Especially, it implies $k(n,m)\leq k(n,m+1)$.

Now we prove two theorems: the first one for $n=2$ and the second one for $n=3$.

#### Theorem 1

*Let* $m\geq2$. *Then there is* $0< k(2,m)< m-1$ *such that*

1. *If* $0\leq k\leq k(2,m)$, *then*
  11 $$\begin{aligned} \bigl(x_{1}^{a}+x_{2}^{a} \bigr)^{m}\geq2^{m-1} \bigl(x_{1}^{a(1+k)}+x_{2}^{a(1+k)} \bigr) \end{aligned}$$
  *for all* $a\geq0$ *and* $0< x_{1},x_{2}$ *such that* $x_{1}x_{2}=1$;
2. *If* $k\geq m-1$, *then*
  12 $$\begin{aligned} \bigl(x_{1}^{a}+x_{2}^{a} \bigr)^{2}\leq2^{m-1} \bigl(x_{1}^{a(1+k)}+x_{2}^{a(1+k)} \bigr) \end{aligned}$$
  *for all* $a\geq0$ *and* $0< x_{1},x_{2}$ *such that* $x_{1}x_{2}=1$;
3. *If* $k(2,m)< k< m-1$, *then for each* $a\geq0$ *there are* $0< x_{1},x_{2}$, $0< y_{1},y_{2}$ *such that* $x_{1}x_{2}=1$, $y_{1}y_{2}=1$ *and*
  13 $$\begin{aligned}& \bigl(x_{1}^{a}+x_{2}^{a} \bigr)^{m}\geq2^{m-1} \bigl(x_{1}^{a(1+k)}+x_{2}^{a(1+k)} \bigr), \end{aligned}$$
  14 $$\begin{aligned}& \bigl(y_{1}^{a}+y_{2}^{a} \bigr)^{m}\leq2^{m-1} \bigl(y_{1}^{a(1+k)}+y_{2}^{a(1+k)} \bigr), \end{aligned}$$
4. $k(2,m)=\sqrt{m}-1$ *for* $m\geq2$.

#### Proof

Put $y=x_{1}^{a}$ and $m=2$ in *F* (see Lemma 3). We can suppose that $0< y\leq1$. Denote

15 $$\begin{aligned} s(y)=y^{4}+2y^{2}+1-2y^{1+k}-2y^{-(1+k)}, \end{aligned}$$

where $0\leq k<\sqrt{2}-1$. First we show $s(y)\geq0$ for $0< y\leq1$ and $0\leq k<\sqrt{2}-1$. We have $s(1)=0$. If we show $s'(y)\leq0$, then $s(y)\geq0$. We get

16 $$\begin{aligned} s'(y)=4y^{3}+4y-2(3+k)y^{2+k}-2(1-k)y^{-k}. \end{aligned}$$

$s'(y)\leq0$ is equivalent to

17 $$\begin{aligned} s_{1}=4y^{3+k}+4y^{1+k}-2(3+k)y^{2+2k}-2(1-k) \leq0. \end{aligned}$$

Because of $s_{1}(1)=0$, it suffices to show that $s'_{1}(t)\geq0$. We have

18 $$\begin{aligned} s'_{1}=4(3+k)y^{2+k}+4(1+k)y^{k}-2(3+k) (2+2k)y^{1+2k}. \end{aligned}$$

$s'_{1}(y)\geq0$ is equivalent to

19 $$\begin{aligned} s_{2}=2(3+k)y^{2}+2(1+k) -(3+k) (2+2k)y^{1+k}\geq0. \end{aligned}$$

From $s_{2}(1)=2-(1+k)^{2}$ we have $k\leq\sqrt{2}-1$. We get also $s_{2}(0)=2(1+k)>0$.

Lemma 3 gives that (19) can be proved only for $k=\sqrt{2}-1$. So it suffices to show

20 $$\begin{aligned} s_{a}=(1+\sqrt{2})y^{2}+1-(2+ \sqrt{2})y^{\sqrt{2}}\geq0. \end{aligned}$$

We have

21 $$\begin{aligned} s'_{a}=2(1+\sqrt{2})y+1-(2+\sqrt{2}) \sqrt{2}y^{\sqrt{2}-1}. \end{aligned}$$

It implies $s'_{a}=0$ only if $y=0$ or $y=1$. Because of $s'_{a}(0.5)=-0.2092$, we deduce $s'_{a}(y)\leq0$ for $0< y<1$. Because of $s_{a}(1)=0$, the proof for $m=2$ is complete. Put

22 $$\begin{aligned} s_{m}(y)= \biggl(\frac{1+y^{2}}{2y} \biggr)^{m^{2}}-\frac{1}{2} \bigl(y^{m}+y^{-m} \bigr). \end{aligned}$$

We have $s_{m}(y)\geq0$ is equivalent to

23 $$\begin{aligned} v_{m}(y)=m^{2}\ln \biggl(\frac{1+y^{2}}{2y} \biggr)-\ln \bigl(y^{m}+y^{-m} \bigr)+\ln(2)\geq0. \end{aligned}$$

It is evident that $v_{m}(1)\geq0$. Next computation gives

24 $$\begin{aligned} v'_{m}(y)=2m\ln \biggl(\frac{1+y^{2}}{2y} \biggr)+\ln(y) \biggl(\frac {1-y^{2m}}{1+y^{2m}} \biggr). \end{aligned}$$

We have

25 $$\begin{aligned} v'_{m}(y,m=1)=2\ln \biggl( \frac{1+y^{2}}{2y} \biggr)+\ln(y) \biggl(\frac {1-y^{2}}{1+y^{2}} \biggr). \end{aligned}$$

We show $v'_{m}(y,m=1)\geq0$. Put $x=y+1/y$ in $\ln(x)\geq (x^{2}-1)/(2x)$, then we obtain

$$\begin{aligned} \ln \biggl(\frac{1+y^{2}}{2y} \biggr)\geq\frac{(1-y^{2})^{2}}{4y(1+y^{2})}. \end{aligned}$$

$v'_{m}(y,m=1)\geq0$ will be done if we prove

26 $$\begin{aligned} 2\frac{(1-y^{2})^{2}}{4y(1+y^{2})}+\ln(y) \biggl(\frac {1-y^{2}}{1+y^{2}} \biggr) \geq0. \end{aligned}$$

It is equivalent to $\ln(y)\geq (\frac{y^{2}-1}{2y} )\geq0$, which is a known formula. Next computation gives

27 $$\begin{aligned} v''_{m}(y)=2\ln \biggl( \frac{1+y^{2}}{2y} \biggr)-\frac{4y^{2m}\ln ^{2}(y)}{(1+y^{2m})^{2}}. \end{aligned}$$

We show again that $v''_{m}(y,m=1)\geq0$. We have

28 $$\begin{aligned} v''_{m}(m=1)=2\ln \biggl( \frac{1+y^{2}}{2y} \biggr)-\frac{4y^{2}\ln ^{2}(y)}{(1+y^{2})^{2}}\geq w(y)=\frac{(1-y^{2})^{2}}{2y}- \frac {4y^{2}\ln^{2}(y)}{1+y^{2}}\geq0. \end{aligned}$$

But $w(y)\geq0$ is evident because this is equivalent to $(1-t)^{2}\geq0$. Next we get

29 $$\begin{aligned} v'''_{m}(y)=- \frac{8\ln^{3}(y)y^{2m}(1-y^{4m})}{(1+y^{2m})^{4}}\geq0. \end{aligned}$$

So $v''_{m}$ is an increasing function. Because of $v''_{m}(m=1)\geq 0$, we obtain $v''_{m}\geq0$ for each $0< y<1$. So $v'_{m}$ is an increasing function. Because of $v'_{m}(m=1)\geq0$, we obtain $v'_{m}\geq0$ for each $0< y<1$. So $s_{m}$ is an increasing function. Because of $s_{m}(m=1)\geq0$, we obtain $s_{m}\geq0$ for each $0< y<1$ and $m\geq1$. So we proved (11).

Next we show that $k=\sqrt{m}-1$ is the best constant. We have

30 $$\begin{aligned} s''_{m}(y) =& 4m(m-1) \bigl(1+y^{2} \bigr)^{m-2}y+2m \bigl(1+y^{2} \bigr)^{m-1}-2^{m-1} \end{aligned}$$

31 $$\begin{aligned} &{} \times(m+1+k) (m+k)y^{m+k-1}-2^{m-1}(m-1-k) (m-2-k)y^{m-3-k}. \end{aligned}$$

We get

32 $$\begin{aligned} s''_{m}(1) = 2^{m-1} \bigl(m-(1+k)^{2} \bigr). \end{aligned}$$

It implies that, for each $m\geq2$ and $k>\sqrt{m}-1$, the function $s_{m}(y)$ is a strictly concave function in some neighborhood $O_{m}(1)$ of 1. So $s_{m}(y)<0$ for some $y<1$ and $y\in O_{m}(1)$. It follows from $s_{m}(1)=0$, $s'_{m}(1)=0$.

Next assertions follow from the previous lemmas and from $\lim_{y\rightarrow0^{+}}s_{m}(y)=+\infty$ for $0\leq k< m-1$. □

#### Note 3

We note that Theorem 1 implies $\lim_{m\rightarrow+\infty }k(2,m)=+\infty$.

#### Theorem 2

*There is* $0< k(3,2)<1$ *such that*

1. *If* $0\leq k< k(3,2)$, *then*
  33 $$\begin{aligned} \bigl(x_{1}^{a}+x_{2}^{a}+x_{3}^{a} \bigr)^{2}\geq3 \bigl(x_{1}^{a(1+k)}+x_{2}^{a(1+k)}+x_{3}^{a(1+k)} \bigr) \end{aligned}$$
  *for all* $a\geq0$ *and* $0< x_{1},x_{2},x_{3}$ *such that* $x_{1}x_{2}x_{3}=1$;
2. *If* $k\geq1$, *then*
  34 $$\begin{aligned} \bigl(x_{1}^{a}+x_{2}^{a}+x_{3}^{a} \bigr)^{2}\leq3 \bigl(x_{1}^{a(1+k)}+x_{2}^{a(1+k)}+x_{3}^{a(1+k)} \bigr) \end{aligned}$$
  *for all* $a\geq0$ *and* $0< x_{1},x_{2},x_{3}$ *such that* $x_{1}x_{2}x_{3}=1$;
3. *If* $k(3,2)< k<1$, *then for each* $a\geq0$ *there are* $0< x_{1},x_{2},x_{3}$, $0< y_{1},y_{2},y_{3}$ *such that* $x_{1}x_{2}x_{3}=1$, $y_{1}y_{2}y_{3}=1$ *and*
  35 $$\begin{aligned}& \bigl(x_{1}^{a}+x_{2}^{a}+x_{3}^{a} \bigr)^{2}\geq3 \bigl(x_{1}^{a(1+k)}+x_{2}^{a(1+k)}+x_{3}^{a(1+k)} \bigr), \end{aligned}$$
  36 $$\begin{aligned}& \bigl(y_{1}^{a}+y_{2}^{a}+y_{3}^{a} \bigr)^{2}\leq3 \bigl(y_{1}^{a(1+k)}+y_{2}^{a(1+k)}+y_{3}^{a(1+k)} \bigr); \end{aligned}$$
4. $0.40< k(3,2)<0.4048$.

#### Proof

Put again $y_{i}=x_{i}^{a}$. From Lemma 1 we have that

$$ F(y_{1},y_{2},y_{3})= (y_{1}+y_{2}+y_{3} )^{2}-3 \bigl(y_{1}^{(1+k)}+y_{2}^{(1+k)}+y_{3}^{(1+k)} \bigr) $$

has extreme values only on *W*, where

$$ W= \biggl\{ (y_{1},y_{2}); 0< y_{1}\leq1, y_{1}\leq y_{2}, y_{2}\leq \frac{1}{y_{1},y_{2}} \biggr\} . $$

We prove that

1. $\lim_{y_{1}\rightarrow0^{+}}F=+\infty$;
2. $\alpha(y)= (2y+\frac{1}{y^{2}} )^{2}-3 (2y^{1+k}+\frac{1}{y^{2+2k}} )\geq0$ for $0< y\leq1$ and for $0\leq k\leq k(3,2)$;
3. $\beta(y)= (y^{2}+\frac{2}{y} )^{2}-3 (y^{2+2k}+\frac {2}{y^{1+k}} )\geq0$ for $0< y\leq1$ and for $0\leq k\leq k(3,2)$,

where $0.40< k(3,2)<0.4048$ and our proof will be done.

Rewriting *F* we obtain

$$ F=\frac{1}{(y_{1}y_{2})^{2}} \bigl[ \bigl(y_{1}^{2}y_{2}+y_{2}^{2}y_{1}+1 \bigr)^{2}-3 (y_{1}y_{2} )^{(1-k)} \bigl( \bigl(y_{1}^{2}y_{2} \bigr)^{(1+k)}+ \bigl(y_{2}^{2}y_{1} \bigr)^{(1+k)}+1 \bigr) \bigr], $$

which implies $\lim_{y_{1}\rightarrow0^{+}}F=+\infty$ for $0\leq k<1$. Now we show $\alpha(y)\geq0$ for $k=0.40$. We have

$$ \alpha(y)= \biggl(2y+\frac{1}{y^{2}} \biggr)^{2}-3 \biggl(2y^{1.4}+\frac {1}{y^{2.8}} \biggr)\geq0 $$

is equivalent to

$$ \alpha_{1}(y)=4y^{6}+4y^{3}+1-6y^{5.4}-3y^{1.2} \geq0. $$

Put $y=u^{5/3}$, we get

$$ \alpha_{1}(u)=4u^{10}+4u^{5}+1-6u^{9}-3u^{2} \geq0. $$

From $\alpha_{1}(1)=0$ it suffices to show

$$ \alpha'_{1}(u)=40u^{9}+20u^{4}-54u^{8}-6u \leq0. $$

We have

$$ \frac{1}{2}\alpha'_{1}(u)\leq \alpha_{2}(u)-7u^{9}+10u^{3}-6. $$

We used $u^{9}\leq u^{8}\leq u^{7}$. Put $u=v^{1/3}$, we get

$$ \alpha_{2}(v)=-7v^{3}+10v-6. $$

Cardano’s formula gives that there are no real roots of $\alpha _{2}(v)=0$ in $(0,1)$. Because of $\alpha_{2}(0.5)=-15/8$, we get $\alpha_{2}(v)\leq0$. So $\alpha(y)\geq0$ for $y\in(0,1)$. Now we show $\beta(y)\geq0$ for $k=0.41$. We have

$$ \beta(y)= \biggl(y^{2}+\frac{2}{y} \biggr)^{2}-3 \biggl(y^{2+2k}+\frac {2}{y^{1+k}} \biggr), $$

which is equivalent to

$$ \beta_{1}(y)=y^{6}+4y^{3}+4-3y^{4.82}-6y^{0.58} \geq0. $$

Put $y=u^{5}$, we get

$$ \beta_{1}(u)=u^{30}+4u^{15}+4-3u^{24.1}-6u^{2.9} \geq0. $$

From $\beta_{1}(1)=0$ it suffices to show

$$ \beta'_{1}(u)=30u^{29}+30u^{14}-72.3u^{23.2}-17.4u^{1.9} \leq0. $$

We have

$$\begin{aligned} \frac{1}{u}\beta'_{1}(u) =& 30u^{27.1}+30u^{12.1}-72.3u^{21.2}-17.4 \\ \leq&\beta_{2}(u) = 30u^{27}+30u^{12}-72.3u^{21.3}-17.4. \end{aligned}$$

We used $u^{12.1}\leq u^{12}$ and $u^{27.1}\leq u^{27}$ and $u^{21.3}\leq u^{21.2}$. Put $u=v^{1/3}$, we get

$$ \beta_{2}(v)=30v^{9}+30v^{4}-72.3v^{7.1}-17.4 \leq\beta _{3}(v)=-42.3v^{8}+30v^{4}-17.4. $$

We used $v^{9}\leq v^{8}\leq v^{7.1}$. Put $q=v^{4}$, we get

$$ \beta_{3}(q)=-42.3q^{2}+30q-17.4. $$

But $\beta_{3}(q)\leq0$. It follows from the following Cardano’s formula which gives that there are no real roots of $\beta_{3}(q)=0$ in $(0,1)$. Because of $\beta_{3}(0)=-17.4$, we get $\beta_{3}(q)\leq0$. So $\beta(y)\geq0$ for $y\in(0,1)$. This completes our proof. □

**Figure 1:**
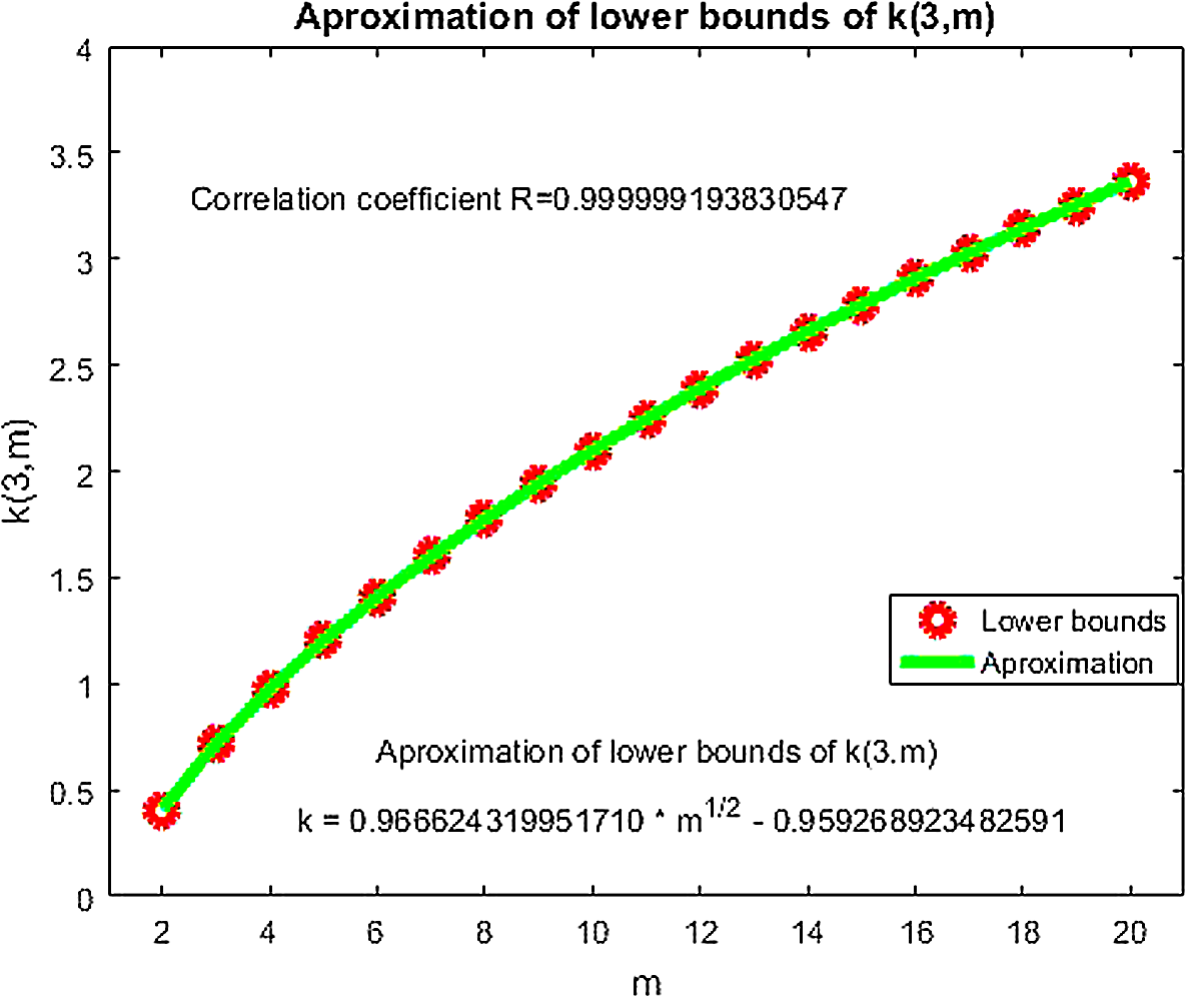
Lower bounds for $n=3$

Now we give the table of bounds of coefficients $k(3,m)$ for $m=2,\ldots,20$. Upper bounds are mathematically proved. It follows from table’s points t in which (11) is not valid (value of $g(t)=F$). The inequality $0.40\leq k(3,2)$ is proved in Theorem 2. The other lower bounds for $k(3,m)$ for $m=3,\ldots,20$ (Table 1) are obtained by MATLAB, so they are not mathematically proved. We made a regression analysis of lower bounds of $k(3,m)$ for $m=2,\ldots,20$ obtained by MATLAB, and our result is described in Fig. 1. We obtained a function $k=0.966624319951710\sqrt {m}-0.959268923482591$ which is a very good approximation of the obtained lower bounds of $k(3,m)$ for $m=2,\ldots,20$. From our lemmas it is evident that $0\leq k(n+1,m)\leq k(n,m)$ and that $\varphi (m)=k(n,m)$ is an increasing function for each fixed $n\in N$, $n\geq 2$. It is also clear that $k(n,m)\leq\sqrt{m}-1$.

**Table 1:** Bounds for coefficients $k(3,m)$ for $m=2,\ldots,20$

*m*	lower bound	upper bound	point t	value of *g*(*t*)
2	0.4047…	0.4048…	0.8151…	−0.000000075…
3	0.7139…	0.7140…	0.8404…	−0.000000041…
4	0.9740…	0.9741…	0.8529…	−0.000002048…
5	1.2028…	1.2029…	0.8632…	−0.000001261…
6	1.4095…	1.4096…	0.8683…	−0.000009898…
7	1.5993…	1.5994…	0.8781…	−0.000037845…
8	1.7760…	1.7761…	0.8806…	−0.000233373…
9	1.9418…	1.9419…	0.8855…	−0.000334923…
10	2.0895…	2.0896…	0.8911…	−0.001123737…
11	2.2475…	2.2476…	0.8966…	−0.001114119…
12	2.3899…	2.3900…	0.8982…	−0.006785876…
13	2.5264…	2.5265…	0.9015…	−0.028610151…
14	2.6577…	2.6578…	0.9051…	−0.023721377…
15	2.7844…	2.7845…	0.9069…	−0.091720248…
16	2.9069…	2.9070…	0.9094…	−0.137691738…
17	3.0257…	3.0258…	0.9098…	−2.510912627…
18	3.1409…	3.1410…	0.9127…	−4.365058865…
19	3.2530…	3.2531…	0.9142…	−15.43949475…
20	3.3622…	3.3623…	0.9153…	−47.82516340…

## Conclusion

In this paper, we made a discussion about a more general inequality than inequality (1). We showed the existence of a function $k(n,m)$ for $n\geq2$, $n\in N$, $m\geq1$ with the following properties:

- If $0\leq k\leq k(n,m)$, then $F\geq0$ is valid for all positive $x_{1},\ldots,x_{n}$ such that $x_{1}\ldots x_{n}=1$;
- If $k(2,m)< k< m-1$, then there are $0< x_{1},\ldots,x_{n}$ such that $x_{1}\ldots x_{n}=1$ and $F\geq0$ is valid, and there are $0< y_{1},\ldots,y_{n}$ such that $y_{1}\ldots y_{n}=1$ and $F\leq0$ is valid;
- If $k>m-1$, then $F\leq0$ is valid for all positive $x_{1},\ldots,x_{n}$ such that $x_{1}\ldots x_{n}=1$.

We also solved the problem 12024-02 published in AMM [2]. Really, if we put $a=10$ and $k=0.3$ in Theorem 2, we obtain inequality (1).

Using Theorem 2 we can get other inequalities which are valid for all positive $x_{1}$, $x_{2}$, $x_{3}$ such that $x_{1}x_{2}x_{3}=1$. For example, we can put $a=10$ and $k=0.4$ and we have

37 $$ \bigl(x^{10}+y^{10}+z^{10} \bigr)^{2}\geq 3 \bigl(x^{14}+y^{14}+z^{14} \bigr), $$

or $a=4$ and $k=0.25$ and we get

38 $$ \bigl(x^{4}+y^{4}+z^{4} \bigr)^{2} \geq3 \bigl(x^{5}+y^{5}+z^{5} \bigr) $$

and so on.

We note that there is an interesting problem: What is equal to $\lim_{n\rightarrow\infty}k(n,m)$ for each fixed $m>1?$
